# Autoimmune Hepatitis in the Setting of Iron Overload Secondary to Heterozygous HFE Gene Mutation

**DOI:** 10.7759/cureus.27614

**Published:** 2022-08-02

**Authors:** Sukhjinder Chauhan, Rasiq Zackria, Deb K Mukhopadhyay

**Affiliations:** 1 Internal Medicine, Sunrise Health Graduate Medical Education Consortium, HCA Healthcare, Mountain View Hospital, Las Vegas, USA; 2 Gastroenterology, Sunrise Health Graduate Medical Education Consortium, HCA Healthcare, Mountain View Hospital, Las Vegas, USA; 3 Gastroenterology and Hepatology, Sunrise Health Graduate Medical Education Consortium, HCA Healthcare, Mountain View Hospital, Las Vegas, USA

**Keywords:** heterozygous hfe gene mutation, dual diagnosis, liver biopsy, hepatology, iron overload, h63d, secondary hemochromatosis, autoimmune hepatitis

## Abstract

Autoimmune hepatitis (AIH) is an inflammatory condition of the liver that is characterized by high titers of certain autoantibodies in the serum. As in other chronic inflammatory conditions, the transferrin saturation in patients with AIH is typically low. However, in rare instances, AIH has been reported to be associated with elevated transferrin saturation secondary to heterozygous *HFE* gene (*H63D*) mutation.

This report describes one such case in which the patient had characteristic histopathologic findings of AIH but was also found to have iron overload and heterozygous *H63D *mutation on genetic testing, leading to the initial dual differential diagnosis of AIH and hemochromatosis, which highlighted the further need of obtaining a liver biopsy.

## Introduction

Autoimmune hepatitis (AIH) is a chronic inflammatory condition typically seen in middle-aged women. It has a variable presentation, including asymptomatic elevation of aminotransferases to extrahepatic symptoms, ranging from fatigue, myalgia, and malaise, to acute liver failure. The diagnosis of AIH is usually established based on the presence of autoantibodies, such as anti-smooth muscle antibodies (ASMA), and the exclusion of other causes of chronic hepatitis, including hemochromatosis or iron overload. However, in rare cases, AIH has been found to be associated with iron overload secondary to heterozygous *HFE* gene mutations. The iron overload can make it challenging to establish an early diagnosis of AIH given both diseases lead to significantly elevated aspartate aminotransferases (AST) and alanine aminotransferases (ALT) at >800s to 1,000s, requiring the need for liver biopsy for definitive diagnoses of AIH, which may result in a delay in the treatment of AIH [1,2].

In this report, we discuss a dual differential diagnosis of AIH and hemochromatosis which were established based on abdominal pain, jaundice on physical examination, abnormal lab results, and serologic iron overload possibly secondary to heterozygous *HFE* gene (*H63D*) mutation identified on genetic testing in a patient who was subsequently diagnosed with AIH with a liver biopsy. After obtaining the liver biopsy results, the patient was started on treatment with azathioprine and prednisone for AIH and was treated via phlebotomy for serologic iron overload.

## Case presentation

A 59-year-old Hispanic woman with a medical history significant for prior stroke without residual deficits, seizure disorder, hypertension, gastroesophageal reflux disease, dyslipidemia, hypothyroidism, and chronic pain presented with dull, non-radiating, left lower quadrant pain for three days. The patient also had multiple episodes of diarrhea and complained of generalized weakness. She denied any history of nausea, vomiting, hematemesis, hematochezia, or melena. Her surgical history was significant for coronary artery bypass graft (CABG) with mitral valve repair and cholecystectomy. She denied alcohol and tobacco use but had been on opioids for chronic back pain. There was no history of blood transfusions and no family history of chronic liver disorders. The patient denied any new medications over the past year. Her home medications included warfarin, levetiracetam, carvedilol, omeprazole, pravastatin, methadone for chronic opioid use disorder, which she developed secondary due to chronic back pain, and levothyroxine. On admission, her vitals were within normal limits. Physical examination revealed tenderness in the left lower quadrant without guarding or rebound. Lab work revealed a normal complete blood count. Results of the comprehensive metabolic profile are shown in Table 1.

**Table 1:** Results of the comprehensive metabolic profile.

Comprehensive metabolic panel	Results (reference)
Total bilirubin	11.4 mg/dL (0.1–1.0 mg/dL)
Direct bilirubin	9.4 mg/dL (0.1–9.2 mg/dL)
Aspartate aminotransferase	960 U/L (15–37 U/L)
Alanine aminotransferase	813 U/L (12–78 U/L)
Alkaline phosphatase	232 U/L (45–117 U/L))
R-factor	10.5 (Value >5 hepatocellular pattern liver injury)
Serum ammonia	131 µ/dL (11–32 µ/dL)
Total protein	6.9 g/dL (6.4–8.3 g/dL)
Albumin	2.3 g/dL (3.4–5.0 g/dL)
Lipase	178 U/L (114–286 U/L)
Prothrombin time	20.6 seconds (9.4–12.5 seconds)
International normalized ratio	1.79 (<1.1 seconds)

Stool studies were positive for *Clostridium difficile* and she was started on oral vancomycin. Computed tomography (CT) of the abdomen and pelvis with contrast demonstrated mild nodularity of the anterior left lobe of the liver surface, reflective of early cirrhosis without evidence of portal hypertension. Due to abnormal liver chemistries and imaging suggestive of liver cirrhosis, iron panel, autoimmune studies, and the viral panel were obtained. The results of these studies are presented in Table 2, Table 3, and Table 4, respectively.

**Table 2:** Results of the iron studies.

Iron studies	Results (reference)
Iron	224 µg/dL (50–170 µg/dL)
Total iron-binding capacity	245 µg/dL (250–450 µg/dL)
Transferrin saturation	93% (20–50%)
Ferritin	1,029 ng/mL (8–252 ng/mL)

**Table 3:** Results of the autoimmune panel.

Immunology (autoimmune panel)	Results (reference)
Immunoglobulin G (Ig)	2,189 mg/dL (586–1,602 mg/dL)
Rheumatoid factor	<10 (Normal <15)
Anti-nuclear antibody (ANA) homogenous pattern	>1:80
ANA atypical speckled pattern	>1:640
Anti-mitochondrial antibodies IgG	<20.0 (0.0–20.0 units)
Anti-smooth muscle IgG antibodies	80 (0–19 units)

**Table 4:** Results of serology. COVID-19: coronavirus disease 2019; Ig: immunoglobulin; CMV: cytomegalovirus; EBV: Epstein-Barr virus

Serology and hepatitis panel	Results (Reference)
COVID-19	Negative
CMV IgM	<30.0 U/mL (0.0–29.9 U/mL)
CMV IgG	<0.60 U/mL (0.0–0.59 U/mL)
EBV IgM	<36.0 U/mL (0.0–35.9 U/mL)
EBV IgG	>600 U/mL (0.0–17.9 U/mL)
Hepatitis B surface antigen	Negative
Hepatitis A IgM antibody	Negative
Hepatitis B core IgM antibody	Negative
Hepatitis C antibody	Negative
Hepatitis C antibody signal/cutoff	0.20 (<0.80)

**Figure 1:**
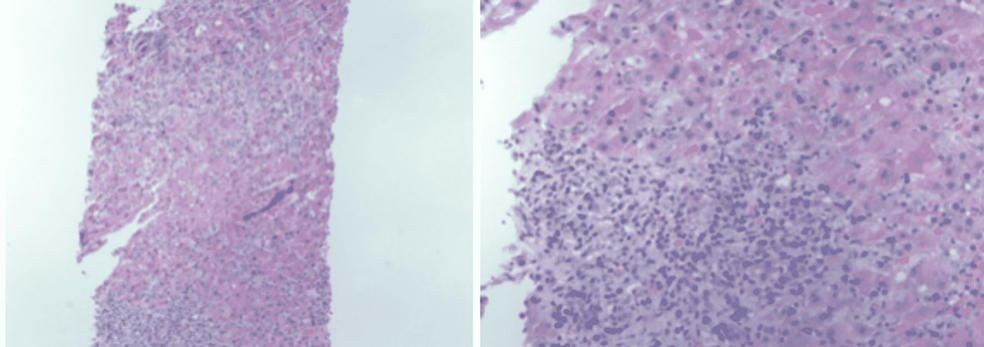
Liver biopsy. Hematoxylin and eosin stain demonstrates moderate chronic inflammatory cell infiltrate composed primarily of lymphocytes with numerous plasma cells suggestive of chronic hepatitis and highly suggestive of autoimmune hepatitis.

These laboratory results suggested a dual differential diagnosis of hemochromatosis and AIH. Genetic testing for *C282Y*, *H63D*, and *S65C *was performed for a definitive diagnosis of hemochromatosis, and the patient was found to have a single copy of the *H63D *gene. The liver biopsy results (Figure 1) were negative for iron staining which ruled out hemochromatosis; however, liver biopsy demonstrated moderate chronic inflammatory cell infiltrate composed primarily of lymphocytes with numerous plasma cells, which was suggestive of AIH.

The patient was started on treatment for AIH with azathioprine 50 mg daily and prednisone 40 mg daily with a tapering strategy. Phlebotomy was recommended for the management of iron overload. The patient was advised to follow up with a gastroenterologist and a hematologist on an outpatient basis for long-term management.

## Discussion

AIH is a chronic inflammatory condition that is around four times more common in women than in men [1,2] and can be associated with other autoimmune diseases [3]. It can affect individuals of any age but most frequently affects middle-aged adults. The estimated prevalence of AIH in the United States is 31.2 per 100,000 [4].

The presentation of AIH varies from asymptomatic elevation of aminotransferases to extrahepatic symptoms, such as fatigue, myalgia, and malaise, to acute liver failure. Diagnosis is based on the presence of autoantibodies, exclusion of other chronic causes of liver disease, and liver histology. AIH management is primarily via immunosuppression therapy, including prednisone with or without azathioprine [5,6]. Based on seropositivity, AIH can be subdivided into two types. Type 1 AIH is the most common type with anti-smooth muscle antibodies (ASMA) and/or anti-nuclear antibodies (ANA), while type 2 AIH is rare with anti-liver/kidney microsomal (anti-LKM) type 1 antibodies and/or anti-liver cytosol (anti-LC) type 1 antibodies [5,7].

Our patient presented with left lower quadrant pain that was subsequently determined to be caused by *Clostridium difficile*. Lab work on admission demonstrated markedly elevated aminotransferases and an R factor of >5 suggesting a hepatocellular pattern of liver injury. The differential diagnoses included acute viral hepatitis, drug-induced liver injury, ischemic liver injury, hemochromatosis, Wilson disease, and AIH. Viral hepatitis, Wilson disease, and ischemic liver injury were ruled out due to normal viral hepatitis panel, absence of neuropsychiatric symptoms and normal ceruloplasmin, and absence of unstable hemodynamic changes. Alcoholic hepatitis was not considered as the patient denied alcohol use, and AST is typically two to three times greater than ALT [8]; however, in our patient, AST to ALT ratio was <2:1, and serum alcohol levels were not obtained as the patient was hospitalized for two days before the gastroenterology consultation. The presence of anti-smooth muscle antibodies pointed toward a diagnosis of AIH; however, in chronic inflammatory conditions, the transferrin saturation is typically low. This occurs because systemic inflammation results in the formation of numerous cytokines, such as interleukin-6 (IL-6), which cause upregulation of liver hepcidin and serum ferritin, while reducing the levels of serum iron and transferrin, which is the iron-transporting protein [9,10]. The iron overload, as indicated by the markedly elevated transferrin saturation of 94%, in our patient could not be explained by a diagnosis of AIH alone as the transferrin saturation is typically low to normal of 20-50% in the setting of chronic inflammatory conditions such as AIH. Given these findings, iron overload (Table 2) could not be explained by a diagnosis of AIH alone and without confirmatory testing raised suspicion of a coexisting condition such as hemochromatosis. This led to a diagnostic dilemma, which required further testing, such as *HFE *genetic testing and liver biopsy. Eventually, a definitive diagnosis of AIH was established based on the liver biopsy results. The diagnosis of primary hemochromatosis was ruled out based on the heterozygous *H63D *gene on genetic testing as primary hemochromatosis requires autosomal recessive gene mutation. Furthermore, secondary hemochromatosis was ruled out based on negative iron staining on the liver biopsy.

*HFE*-related hereditary hemochromatosis is an autosomal recessive disorder, and most patients are homozygous for the *C282Y *mutation. However, the presence of the heterozygous *C282Y *mutation has also been linked with an increased hepatic concentration of iron in patients with chronic liver diseases, such as AIH. In a German study, 17.2% of patients diagnosed with AIH were found to have the heterozygous *C282Y *mutation [11]. On the other hand, heterozygosity of *H63D *gene mutation causing iron overload in patients with AIH has rarely been reported in the literature. To our knowledge, this is the second such case to be reported, the first reported by Bollimunta et al. [12]. The exact pathophysiology behind the elevated transferrin saturation in patients with AIH remains unclear. However, possible mechanisms may include dysregulation of iron absorption in enterocytes and the release of iron from hepatocytes following necro-inflammatory cell damage [13].

It is important to note that heterozygosity for the *HFE* gene mutation is rarely associated with significant inflammatory liver damage and fibrosis due to iron overload alone [1,10]. However, it can create a diagnostic dilemma and result in significant diagnostic delays. This case emphasizes the importance of liver biopsy and genetic testing in diagnosing AIH in patients who have both AIH and serologic iron overload findings.

## Conclusions

AIH refers to the chronic inflammation of the liver, which may present with chronic hepatitis or fulminant liver failure. The diagnosis of AIH is usually established based on the presence of autoantibodies and by excluding other causes of chronic hepatitis. Transferrin saturation is typically low in patients with AIH and this distinguishes it from hemochromatosis, which is another cause of chronic hepatitis. However, in a few patients, AIH has been found to be associated with high transferrin saturation, which can pose a diagnostic dilemma in the setting of new-onset hepatitis. The delay in diagnosis can result in a subsequent delay in the initiation of treatment and may have serious implications for the patient. Therefore, physicians should have a low threshold for performing a liver biopsy and genetic testing to confirm the diagnosis in patients who have features of both AIH and iron overload.
